# Promethearchaeum syntrophicum gen. nov., sp. nov., an anaerobic, obligately syntrophic archaeon, the first isolate of the lineage ‘Asgard’ archaea, and proposal of the new archaeal phylum Promethearchaeota phyl. nov. and kingdom Promethearchaeati regn. nov.

**DOI:** 10.1099/ijsem.0.006435

**Published:** 2024-07-05

**Authors:** Hiroyuki Imachi, Masaru K. Nobu, Shingo Kato, Yoshihiro Takaki, Masayuki Miyazaki, Makoto Miyata, Miyuki Ogawara, Yumi Saito, Sanae Sakai, Yuhei O. Tahara, Yoshinori Takano, Eiji Tasumi, Katsuyuki Uematsu, Toshihiro Yoshimura, Takashi Itoh, Moriya Ohkuma, Ken Takai

**Affiliations:** 1 Institute for Extra-cutting-edge Science and Technology Avant-garde Research (X-star), Japan Agency for Marine-Earth Science and Technology (JAMSTEC), Yokosuka, Japan; 2 Japan Collection of Microorganisms (JCM), RIKEN BioResource Research Center, Tsukuba, Japan; 3 Submarine Resources Research Center, JAMSTEC, Yokosuka, Japan; 4 Graduate School of Science, Osaka Metropolitan University, Osaka, Japan; 5 Biogeochemistry Research Center, JAMSTEC, Yokosuka, Japan; 6 Department of Marine and Earth Sciences, Marine Work Japan, Yokosuka, Japan; 7 Section for Exploration of Life in Extreme Environments, Exploratory Research Center on Life and Living Systems (ExCELLS), National Institute of Natural Sciences, Okazaki, Japan

**Keywords:** ‘Asgard’ archaea, anaerobe, deep-sea sediment, *Promethearchaeum*, syntrophy

## Abstract

An anaerobic, mesophilic, syntrophic, archaeon strain MK-D1^T^, was isolated as a pure co-culture with *Methanogenium* sp. strain MK-MG from deep-sea methane seep sediment. This organism is, to our knowledge, the first cultured representative of ‘Asgard’ archaea, an archaeal group closely related to eukaryotes. Here, we describe the detailed physiology and phylogeny of MK-D1^T^ and propose *Promethearchaeum syntrophicum* gen. nov., sp. nov. to accommodate this strain. Cells were non-motile, small cocci, approximately 300–750 nm in diameter and produced membrane vesicles, chains of blebs and membrane-based protrusions. MK-D1^T^ grew at 4–30 °C with optimum growth at 20 °C. The strain grew chemoorganotrophically with amino acids, peptides and yeast extract with obligate dependence on syntrophy with H_2_-/formate-utilizing organisms. MK-D1^T^ showed the fastest growth and highest maximum cell yield when grown with yeast extract as the substrate: approximately 3 months to full growth, reaching up to 6.7×10^6^ 16S rRNA gene copies ml^−1^. MK-D1^T^ had a circular 4.32 Mb chromosome with a DNA G+C content of 31.1 mol%. The results of phylogenetic analyses of the 16S rRNA gene and conserved marker proteins indicated that the strain is affiliated with ‘Asgard’ archaea and more specifically DHVC1/DSAG/MBG-B and ‘Lokiarchaeota’/‘Lokiarchaeia’. On the basis of the results of 16S rRNA gene sequence analysis, the most closely related isolated relatives were *Infirmifilum lucidum* 3507LT^T^ (76.09 %) and *Methanothermobacter tenebrarum* RMAS^T^ (77.45 %) and the closest relative in enrichment culture was *Candidatus* ‘Lokiarchaeum ossiferum’ (95.39 %). The type strain of the type species is MK-D1^T^ (JCM 39240^T^ and JAMSTEC no. 115508). We propose the associated family, order, class, phylum, and kingdom as *Promethearchaeaceae* fam. nov., *Promethearchaeales* ord. nov., *Promethearchaeia* class. nov., *Promethearchaeota* phyl. nov., and *Promethearchaeati* regn. nov., respectively. These are in accordance with ICNP Rules 8 and 22 for nomenclature, Rule 30(3)(b) for validation and maintenance of the type strain, and Rule 31a for description as a member of an unambiguous syntrophic association.

## Introduction

Members of ‘Asgard’ archaeal lineage have been identified as the prokaryotic entities most closely related to eukaryotes, as evidenced by their phylogenetic position and the abundance of eukaryotic signature proteins (ESPs) in their genomes [1,5]. Consequently, this archaeal group has garnered significant interest as a key prokaryote group for elucidating the origin of eukaryotes. Additionally, these archaea are also known to be an important microbial group in the global biogeochemical cycle, as they are frequently detected in marine sediments [6,7].

Recently, we successfully isolated strain MK-D1^T^the first cultured representative of ‘Asgard’ archaea/‘Asgardarchaeota’, as a pure syntrophic co-culture with *Methanogenium* sp. strain MK-MG, from deep-sea sediment of the Nankai Trough at a water depth of 2533 m, off the Kumano area, Japan [8]. The co-culture was obtained through a 12 year cultivation effort by a combination of a continuous-flow bioreactor system and conventional batch cultivation techniques [8,10]. In our previous report, we tentatively named the strain *Candidatus* (*Ca*.) ‘Prometheoarchaeum syntrophicum’ [8]. Here, we report the strain’s detailed physiological properties and propose the name *Promethearchaeum syntrophicum* gen. nov., sp. nov. to accommodate the strain, as well as the associated family, order, class, phylum, and kingdom names in accordance with the current International Code of Nomenclature of Prokaryotes (ICNP) rules [11,14].

## Methods

### Isolation and cultivation

The syntrophic pure co-culture of MK-D1^T^ and *Methanogenium* sp. strain MK-MG was isolated from an anaerobic methane-oxidizing microbial community in a continuous-flow down-flow hanging sponge (DHS) reactor [8]. The bioreactor was inoculated with sediment from a methane seep at the Omine Ridge, Nankai Trough, off the Kumano area, Japan (33° 7.2253′ N, 136° 28.6672′’ E), 2533 m below sea level. The detailed sediment sample/site information [15,16] and enrichment/isolation procedures have been described previously [8,10]. The culture media used in this study was prepared according to methods described in a previous report [10]. After establishment of the pure co-culture, all subsequent cultures were incubated at 20 °C in 50 ml serum vials containing 20 ml basal medium supplemented with casamino acids (0.05 %, w/v) and powdered milk (0.05 %, w/v) or only yeast extract (0.05 %, w/v), unless otherwise stated. Sub-cultures were prepared using an inoculum volume amounting to 5–20 % of the medium volume. The purity of the cultures was confirmed as described previously [8]. For the pure co-culture of MK-D1^T^ with *Halodesulfovibrio* sp. strain MK-HDV, purity was confirmed by 16S rRNA gene tag-sequencing analysis (Table S1, available in the online version of this article).

### Microscopy

Cell morphology was observed using extreme high-resolution focused ion beam-scanning electron microscopy (FIB-SEM), transmission electron microscopy (TEM), and cryo-electron microscopy on MK-D1 cells grown in casamino acids-powdered milk medium, as described previously [8].

For FIB-SEM, cells were fixed in casamino acid–powdered milk medium with glutaraldehyde [2.5 % (w/v) final concentration] for 2 h at 20 °C, followed by overnight fixation at 4 °C. After washing in the anaerobic basal medium (excluding organic substances), cell suspensions were adhered to the poly-l-lysine (Sigma) coated glass slides overnight at 4 °C. Following this treatment, the cells were fixed in 2 % (w/v) osmium tetroxide dissolved in the anaerobic basal medium. After the samples were rinsed with distilled water, conductive staining was performed by incubation in 0.2 % aqueous tannic acid (pH 6.8) for 30 min; the samples were then washed with distilled water and treated with 1 % aqueous osmium tetroxide for 30 min. After that, the samples were dehydrated in a graded ethanol series and critical-point dried in a JCPD-5 instrument (JEOL). The samples were coated with osmium using an osmium plasma coater (OPC80; Filgen) and observed with FIB-SEM (Helios G4 UX, ThermoFisher Scientific) operated at 1 kV.

For TEM, cells were prefixed in casamino acid–powdered milk medium with 2.5 % (w/v) glutaraldehyde for 2 h at 20 °C. The specimens were frozen in a high-pressure freezing apparatus (EM-PACT2, Leica). The frozen samples were substituted with 2 % OsO_4_ in acetone for 3–4 days at −80 °C. Then the samples were warmed gradually to 4 ℃, *en* bloc stained with 1 % uranyl acetate, rinsed with acetone and embedded in epoxy resin (TAAB). Thin sections (70 nm) were cut with am ultramicrotome (EM-UC7, Leica). Ultrathin sections of the cells were stained with 2 % uranyl acetate and lead-stained solution (0.3 % lead nitrate and 0.3 % lead acetate, Sigma-Aldrich), and were observed using TEM (Tecnai 20, FEI) at an acceleration voltage of 120 kV.

The architecture of the cellular envelope was examined by employing the quick-freeze deep-etch replica electron microscopy (QFDE-EM) method. For this, four aliquots of 450 ml cultures were concentrated to approximately 6.5 ml through filtration using a 0.22 µm pore size polyethersulfone filter unit (Corning) and finally transferred to a glass vial in an anaerobic chamber [95 : 5 (v/v) N_2_:H_2_ atmosphere; COY Laboratory Products]. To remove the H_2_ (inhibitory to the strain), this vial’s headspace was replaced with N_2_/CO_2_ gas (80 : 20, v/v) via flushing. Just before the quick-freezing step, the above culture liquid was further concentrated in a plastic tube to approximately 100 µl by centrifugation at 20 400 ***g*** for 10 min at 20 °C and removal of the supernatant. Subsequently, 10 µl of the concentrated liquid culture samples were frozen in liquid helium using a CryoPress (Valiant Instruments). The samples were then processed according to the protocol of Tahara and Miyata [17], and the replica samples were observed by using TEM (JEM-1010, JEOL).

### Phenotypic characterisation

Due to the low cell yield, growth of MK-D1^T^ was determined by quantitative polymerase chain reaction (qPCR) as described previously [8]. Substrate utilisation of the strain was examined in basal medium supplemented with each substrate sterilised via autoclave or filtration. Casamino acids (0.0005 %, w/v) and 20 proteinogenic amino acids (1 µM each) were supplemented at low concentrations when testing growth on non-protein-derived substrates, as MK-D1^T^ lacks the capacities to synthesise most amino acids and vitamins [8]. Note that we have previously demonstrated that MK-D1^T^ grows minimally on casamino acids and the 20 proteinogenic amino acid mixture at the above concentrations (the 16S rRNA gene copy number changed less than one order of magnitude) [8]. For media supplemented with H_2_ or formate, 2-bromoethansulfonate (2-BES), a methanogenesis inhibitor, was added at a final concentration of 10 mM. All the incubations were performed in duplicate for 120 days at 20 °C in the dark without shaking. Growth on each substrate was determined by qPCR. Substrate concentrations (*i.e*., consumption) were measured in subcultures that showed a 16S rRNA gene copy number increased by tenfold or more. Here, we report utilisation of substrates by MK-D1^T^ only for those which supported growth (an increase of tenfold or greater) and for which clear consumption was detectable. Note that measurement of substrate concentrations was not performed for complex substrates, such as powdered milk, peptone, casein digest, and yeast extract. Amino acid concentrations were quantified by gas chromatography as described previously [8]. Monosaccharide concentrations were measured by high-performance liquid chromatography as described previously [18]. Organic acids were measured using the ion-exclusion chromatography mode of the 930 Compact IC Flex (Metrohm) using a Metrosep Organic Acids - 250/7.8 column without a chemical suppressor module. Concentrations of sulphate, thiosulphate, and nitrate were determined by ion-exchange chromatography with electrical conductivity detection, on the same 930 Compact IC Flex system (Metrohm), as described previously [19].

Evaluation of growth temperature was performed using a basal medium containing casamino acids and powdered milk. The cultures were incubated at 4, 10, 15, 20, 25, 30, 37, and 40 °C. The effects of NaCl concentration on growth were determined with 5, 10, and 20 g l^−1^ in the basal medium. Effects of antibiotics on growth were evaluated by supplementing the medium with each antibiotic at a final concentration of 100 µg ml^−1^ (except for chloramphenicol, which was amended to 50 µg ml^−1^). All incubations were performed in triplicate. For the roll-tube method, solid medium was prepared by adding purified agar (Agar Noble, Difco) to the basal medium at a final concentration of 20 g l^−1^.

Lipid analysis was performed as described previously [8]. Because *Methanogenium* sp. strain MK-MG could not be isolated in an axenic culture, *Methanogenium cariaci* strain JR1^T^ was used as a reference organism in the lipid analysis. The strain was purchased from JCM. (*i.e*., JCM 10550^T^) and cultured in the basal medium supplemented with H_2_ (approximately 150 kPa in the headspace of the culture bottle) and acetate (1 mM) and yeast extract (0.01 %, w/v) at 20 °C in the dark.

### Genome sequencing and phylogenetic analysis

DNA extraction was performed as described previously [18]. The paired-end library was constructed with KAPA Hyper Prep Kit (Illumina) platforms according to the manufacturer’s instructions. Library sequencing was performed using the MiSeq platform (Illumina; 2×300 bp). Raw Illumina reads were sequentially processed with Trimmomatic version 0.39 [20] and assembled using CLC Genomics Workbench version 23.0.4 (Qiagen) with the following parameter settings: 64 bp word size, 500 bp bubble size, map read back to the contigs with 0.9 length fraction and a 0.9 similarity fraction. The genomes of MK-D1^T^ and MK-MG were binned on the basis of differences in the contigs with respect to DNA G+C content and sequence coverages. To obtain a complete genome sequence for MK-D1^T^, contigs in the MK-D1 bin were ordered by reference-guided approaches to the previously deposited *Candidatus*. ‘P. syntrophicum’ MK-D1 genome (GenBank accession number CP042905) and gaps were filled using the GapCloser programme implemented in the SOAPdenovo package [21]. Coding sequences (CDSs) in the genome were predicted using Prodigal v2.6.3 [22,23]. The deduced amino acid sequences were subjected to a BLASTP search against the National Centre for Biotechnology Information non-reduntant proteins database (NCBI–nr) and Kyoto Encyclopedia of Genes and Genomes (KEGG) GENES databases, and their functional annotations were manually assigned based on the KEGG Orthology database. The prediction of rRNA and non-coding RNA genes were performed with Rfam v13.0 [23]. The tRNA genes were searched using tRNAscan-SE v1.3.1 [24].

A maximum-likelihood tree based on 16S rRNA gene sequences was reconstructed using representative sequences collected from silva release 138.1 [25] under the phylum-equivalent category ‘Asgardarchaeota’ and GenBank nucleotide sequences and genome assemblies under the ‘Asgard’ group clustered with a minimum sequence identity threshold of aligned 90 % using CD-HIT v4.8.1 [26]. Sequences were aligned using MAFFT v7.49 (--maxiterate 1000 --localpair) [27]. A maximum-likelihood tree was reconstructed using IQTREE v2.2.6 [28] using an optimal model chosen using ModelFinder (-m MFP) and 100 bootstrap replicates (-b 100). Bootstrap values were recalculated using BOOSTER [29].

Maximum-likelihood estimation of a phylogenetic tree was also performed using a concatenated alignment of translation- and transcription-related proteins conserved among most archaea – a subset of the GTDB r214 [30] marker proteins excluding marker proteins of other function (TIGR00064, TIGR00522, and PF04919.13) and marker proteins that were not detected in at least 20 % of the genomes included in the analysis (TIGR01502, TIGR01171, TIGR02236, TIGR03627, TIGR03629, and TIGR03673). Species representative genomes for the GTDB r214 [30] phylum-equivalent group ‘Asgardarchaeota’ and type species for each class of the phyla *Thermoproteota*, *Halobacteriota*, and *Methanobacteriota* were included. Marker proteins were aligned using MAFFT v7.49 (--maxiterate 1000 --localpair) [27], concatenated, and trimmed using Block Mapping and Gathering with Entropy (BMGE) v1.12 [31] with the Blocks Substitution Matrix with less than 30 % similarity (BLOSUM30) and maximum gap rate of each position of 0.67 and minimum length of selected regions of 3. Maximum-likelihood estimation was performed using IQ-TREE v2.2.6 [28] with the universal distribution mixture (UDM) model with 64 components and log centred log-ratio (LCLR) transformation [32] and 1000 ultrafast bootstrap replicates (-B 1000). Bootstrap values were recalculated using BOOSTER [29].

For calculation of evolutionary distances between archaeal lineages, aligned 16S rRNA sequences were obtained from silva r138.1 [25]. Sequences longer than 1100 bases with sequence, alignment, and pintail qualities greater than 80 % were retained and further clustered based on 97 % sequence identity using CD-HIT v4.8.1 [26]. Distances between the representative sequences were calculated using the distmat function of EMBOSS v6.6.0 [33]. Sequences belonging to class *Thermoplasmata* of the phylum *Thermoplasmatota *and the class *Halobacteria* of the phylum *Halobacteriota *were omitted from downstream calculations as these lineages had extraordinarily high distances from other lineages (as reflected by long branches at the base of the two lineages) that would skew the analyses. The distances between the class *Thermoplasmata* and other classes of the kingdom *Methanobacteriati* were significantly greater than distances between non-*Thermoplasmata* classes of the kingdom *Methanobacteriati* (30.26±1.39 and 26.90±1.84 substitutions per 100 bases; *P*<0.05). Likewise, the distances between the class *Halobacteria* and other classes of the phylum *Halobacteriota* were significantly greater than distances between non-*Halobacteria* classes of the phylum *Halobacteriota* (29.34±1.64 and 23.73±1.43; *P*<0.05). Statistical significance (*P*-values) was calculated using Student’s *t*-test.

## Results and discussion

### Cell morphology

As reported previously [8], cells of MK-D1^T^ were small cocci (diameters of 300–750 nm with an average of 550 nm) and typically formed aggregates surrounded by extracellular polymer substances (EPS) during exponential growth. The cells produce membrane vesicles (50–280 nm in diameter) and chains of blebs [8]. The cells also formed protrusions of uniform diameter (80–100 nm) and various lengths that were linear and often branching [8] (Fig. S1). In cultures incubated for more than 105 days, we found that the vast majority of cells had membrane-based protrusions and spherical cells lacking protrusions were uncommon. This pattern indicates that the formation of protrusions probably occurs during the late exponential phase. Here, we further observed that cells could have up to eight protrusions and some protrusions had bulging sections and rosary-like connections between bulges (Fig. S1d, i). Dividing cells had no protrusions, and they had less EPS and a ring-like structure around the cells (Fig. S1f, g). Although MK-D1^T^ possesses homologues of surface layer (S-layer) related proteins [8], QFDE-EM showed that the cell surface of MK-D1^T^ lacks the regular structures frequently observed in other microbial cells that possess an S-layer (Fig. S1a–g) [34,35].

### Physiology

MK-D1^T^ was strictly anaerobic and required the presence of reducing agents such as sodium sulphide and cysteine–HCl. MK-D1^T^ could grow anaerobically on protein/peptide-containing substrates through syntrophic association with methanogenic archaea and sulphate-reducing bacteria via interspecies hydrogen/formate transfer [8]. Specifically, growth was observed on the following substrates: casamino acids (0.05 %, w/v), powdered milk (0.01 %, w/v), casein digest (0.05 %, w/v), peptone (0.05 %, w/v), tryptone (0.05 %, w/v), and yeast extract (0.05 %, w/v). Growth on casamino acids alone was unstable (*i.e*., some incubations showed no growth), but it was stable when a mixture of 20 proteinogenic amino acids (0.1 mM) was added. This instability may be due to the lack of tryptophan [36], which MK-D1^T^ cannot synthesise [8], and the consequent need to acquire the compound from its syntrophic partner. Notably, MK-D1^T^ could not grow when provided with a mixture of 20 proteinogenic amino acids (0.1 mM or 0.25 mM each). However, given that MK-D1^T^ has been confirmed to utilise amino acids previously [8] (Table S2), MK-D1^T^ may only be able to utilise exogenous free amino acids in the presence of a substrate that supports growth. This reflects the complex composition of all substrates that supported growth (*i.e*., containing both amino acids and other organic substances). The following substrates were not utilised (1 mM final concentration unless otherwise indicated): H_2_ (approximately 1.5 kPa in the headspace), formate, sugars (i.e., glucose, fructose, xylose, ribose, and maltose), lactate, acetate, citrate, pyruvate, and fumarate. In addition, no growth was observed with alginate (0.01%, w/v) and l-hydroxyproline (1 mM), which were postulated as potential energy sources on the basis of the results of the genomic analysis [8]. MK-D1^T^ did not utilise the following inorganic substances (each at a final concentration at 500 µM) as electron acceptors: sulphate, thiosulphate, and nitrate. This was consistent with the absence of genes associated with the reduction of these substances (*e.g*., *dsr*AB and *nar*G) from the genome.

Growth of MK-D1^T^ on casamino acids–powdered milk medium was very slow, usually requiring a 30–60 day lag phase and more than 3 months to reach full growth with a doubling time of approximately 14–25 days [8]. In addition, the cell yield was extremely low with maximum cell densities of around 9×10^5^ 16S rRNA gene copies ml^−1^ [8]. A reevaluation of the genomic data for potential growth enhancement indicated that MK-D1^T^ cannot synthesise tetrahydrofolate and pantoate, a precursor of coenzyme A [37]. In cultures individually supplemented with either compound, only addition of pantoate increased growth, reaching 1.2×10^6^ 16S rRNA gene copies ml^−1^ (Fig. S2a). After various attempts to further improve proliferation, we found that MK-D1^T^ could reach higher yields (the highest value recorded was 6.7×10^6^ 16S rRNA gene copies ml^−1^) when grown with yeast extract as the substrate and supplemented with pantoate. While lag phase and full growth required similar time spans compared with growth with casamino acids and powdered milk as substrates (*i.e*., 30–60 days and about 3 months, respectively), the doubling time was slightly shorter, approximately 11–24 days (Fig. S2b). Despite the increased growth, yeast extract did not support colony formation of the strain (monitored for 1 year).

MK-D1^T^ grew at temperatures between 10 and 30 °C with optimum growth at 20 °C, as reported previously [8]. We also confirmed that this strain could grow at 4 °C, which is close to the *in situ* temperature of the deep-sea sediment we used in this study (*i.e*., 2 °C), through over 693 days of incubation (Fig. S2c). MK-D1^T^ is a typical marine microorganism, because it did not grow when NaCl concentrations in the medium were reduced to 5 or 10 g l^−1^.

The syntrophic partner of MK-D1^T^ could be replaced. It was shown previously that MK-D1^T^ could also grow syntrophically with *Methanobacterium* sp. strain MO-MB1 (JCM 18473) instead of *Methanogenium* sp. strain MK-MG [8]. However, after several successive transfers of this syntrophic culture, *Methanbacterium* sp. strain MO-MB1 was out-competed and replaced by *Methanogenium* sp. strain MK-MG as the syntrophic partner of MK-D1^T^. This may be due to the low concentrations of H_2_ and formate produced by MK-D1^T^, which favour methanogens with a higher substrate affinity for H_2_/formate, such as *Methanogenium* [38] (Fig. S1j). On the other hand, by adding sulphate (5 mM), 2-BES (10 mM), and a sulphate-reducing bacterium *Halodesulfovibrio* sp. strain MK-HDV (JCM 32479) to casamino acids-powdered milk medium, we could establish a pure co-culture in which *Methanogenium* sp. MK-MG was replaced by *Halodesulfovibrio* sp. strain MK-HDV (Fig. S1k and Table S1). The maximum cell densities in the pure co-culture were 8.0×10^5^–3.5×10^6^ 16S rRNA gene copies ml^−1^ (Table S1), which were similar to those in co-cultures with *Methanogenium* sp. MK-MG in yeast extract medium.

MK-D1^T^ tolerated ampicillin, vancomycin, kanamycin, streptomycin, tetracycline, and erythromycin. Chloramphenicol inhibited growth.

### Chemotaxonomy

As reported previously [8], analysis of lipid composition in the co-culture of MK-D1^T^ and *Methanogenium* sp. strain MK-MG showed the presence of typical archaeal isoprenoids, specifically C_20_-phytane and C_40_-biphytanes with 0–2 cyclopentane rings. Given that many archaeal species and *M. cariaci* strain JR1^T^, which is a close relative of *Methanogenium* sp. strain MK-MG with 99.3 % 16S rRNA gene identity, contained C_20_-phytane and C_40_-biphyane with 0 rings [8,39], MK-D1^T^ probably had C_20_-phytane and C_40_-biphytane with 0–2 rings. Corroborating these observations, the MK-D1 genome encodes the enzymes necessary for synthesising ether-bound lipids with the capability of cyclisation of biosynthesis, with the exception of the gene coding for geranylgeranylglyceryl phosphate synthase.

### Storage

MK-D1^T^ could be stored at 4 °C for longer storage (it has been confirmed that it is able to revive it after 7 months storage). Cultures could be preserved with glycerol (10 % final concentration, v/v) in glass vials sealed under anaerobic conditions (100 % N_2_ in the headspace) at −80 °C. The process of glycerol storage and revival was repeated twice, and it was confirmed that MK-D1^T^ grew after 90 days of incubation using qPCR (Fig. 2d). Other cryoprotectants were not tested.

### Genomic features and phylogeny

The genomes were re-sequenced and re-assembled for the pure co-culture. The genome of MK-D1^T^ included a chromosome of 4 324 194 bp with a DNA G+C content of 31.1 mol%. The genome encoded 3831 predicted proteins, one copy of each of the 5S, 16S, and 23S rRNA genes, and 46 tRNAs. The genome of MK-D1^T^ encoded genes for ten amino acid degradation pathways [8], most of which theoretically require H_2_/formate-scavenging syntrophic interactions on the basis of previous thermodynamic calculations [40]. The genome also contained and expressed ESPs that were also observed in related ‘Asgard’ archaea, such as actin, ubiquitin, and ESCRT-III proteins [8].

**Fig. 1.**
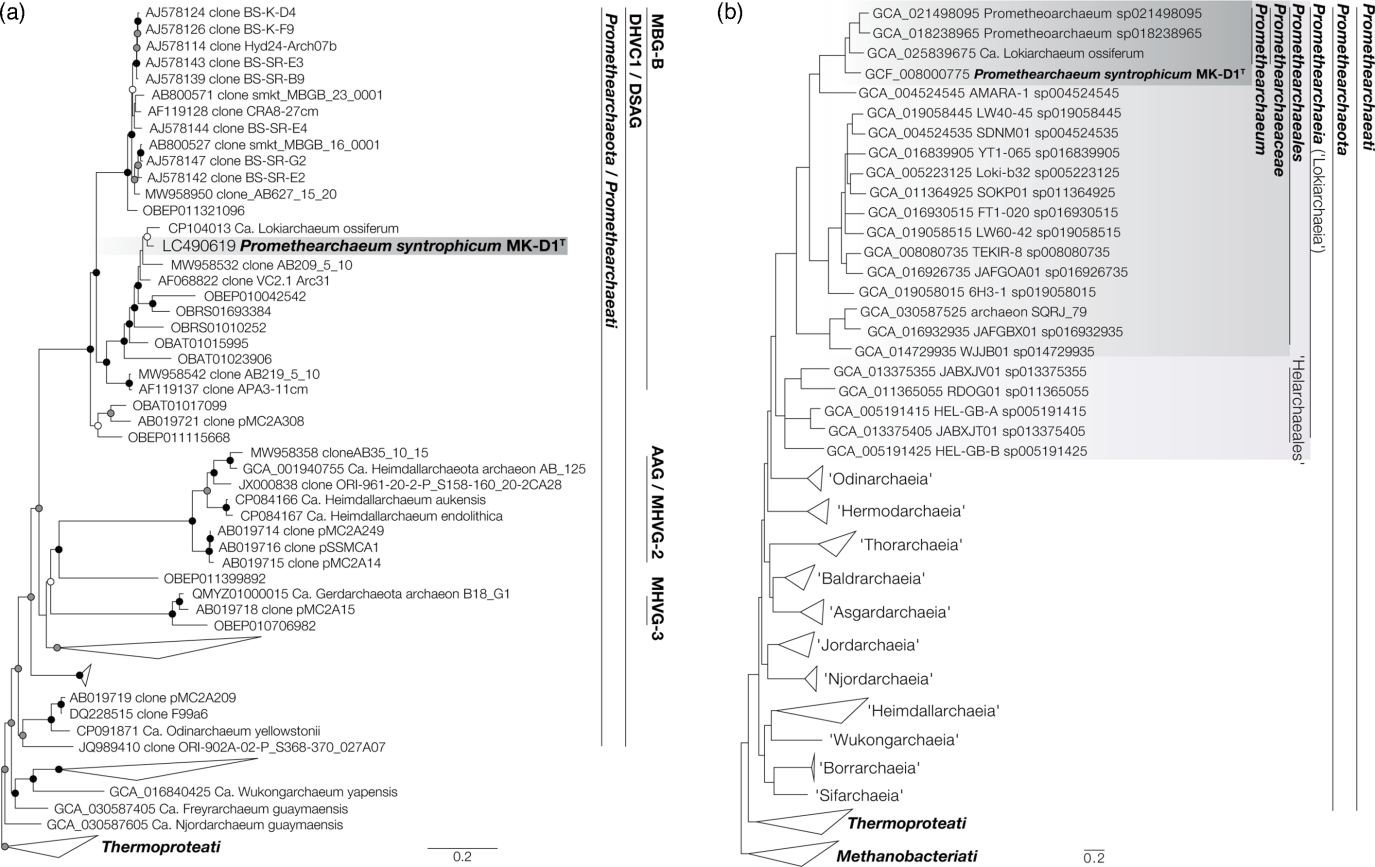
Phylogeny of *Promethearchaeum syntrophicum* MK-D1^T^ based on 16S rRNA gene and conserved marker proteins. (**a**) Maximum-likelihood estimation of the phylogeny of the ‘Asgard’ archaea group based on an alignment of the 16S rRNA gene sequences collected from members of the clade ‘Asgardarchaeota’ in silva release 138.1 and the ‘Asgard’ group in Genbank and clustered with a threshold of 90 % sequence identity. Sequences from type species of classes of the phylum *Thermoproteota* were also included as an outgroup. IQTREE v2.2.6 was used for tree reconstruction with an optimal model chosen by ModelFinder [the symmetrical model with six rate categories (SYM+R6) was chosen], 100 bootstrap replicates and BOOSTER-recalculated bootstrap values. Branch supports are indicated with the following symbols: black circles for ≥90 %, grey for ≥80 %, and white for ≥70 %. Distantly related clades are collapsed into groups (triangles). Approximate ranges of clades previously defined based on 16S rRNA genes are shown. (**b**) Maximum-likelihood estimation of the phylogeny of the ‘Asgard’ archaea group based on a concatenated alignment of a subset of conserved marker proteins defined in GTDB r214. The genus- and species-level classification according to GTDB r214 is shown for each genome. Type species (or representative species for uncultured clades) for classes of the phylum *Thermoproteota*, orders of the phylum *Halobacteriota*, and orders of the phylum *Methanobacteriota* are included as outgroups. IQTREE v2.2.6 was used for tree reconstruction with the universal distribution mixture (UDM) model with 64 components and LCLR transformation, 1000 ultrafast bootstrap replicates, and BOOSTER-recalculated bootstrap values. Branches with low support (<95 %) are collapsed. The same trees with expanded clades are shown in Fig. S3.

No isolated species showing more than 77.45 % 16S rRNA gene identity with MK-D1^T^ were found in the public databases. Therefore, we concluded that it is appropriate to describe MK-D1^T^ as an organism representing a novel genus and species. As consistent with our previous report [8], we confirmed that MK-D1^T^ was affiliated with an archaeal phylogenetic group originally termed Deep-sea Hydrothermal Vent Crenarchaeotic Group (DHVC1) [41], Marine Benthic Group-B (MBG-B) [42], and Deep-sea Archaeal Group (DSAG) [43] and later proposed to be a phylum-level lineage (on the basis of phylogenomic criteria) referred to as ‘Asgardarchaeota’ [44,45] (Figs 1 and S3). This group was previously considered a superphylum-level lineage, referred to as ‘Asgard’ archaea [2], spanning multiple uncultured phylum-level lineages including ‘Lokiarchaeota’ [1], which is now referred to as a class-level lineage ‘Lokiarchaeia’ [46]. The results of phylogenetic analyses based on the 16S rRNA gene (Fig. 1a) and conserved marker proteins (Fig. 1b) also indicate that MK-D1^T^ is a member of an archaeal lineage corresponding to ‘Lokiarchaeia’, DHVC1, MBG-B, and DSAG.

Although, on the basis of the maximum scores of blastn against 16S rRNA gene sequences of cultured organisms, the closest isolated relatives were *Infirmifilum lucidum* 3507LT^T^ (76.09 %) and *Methanothermobacter tenebrarum* RMAS^T^ (77.45 %), a highly enriched culture of a much more closely related archaeon *Ca*. ‘Lokiarchaeum ossiferum’ Loki-B35 (95.39 %) has been reported in a recent study [47]. They are also closely related in terms of average amino acid identity (58.72 %). Like MK-D1^T^, the enrichment culture of *Ca*. ‘L. ossiferum’ has been reported to degrade amino acids/peptides anaerobically under mesophilic conditions [47]. Whether *Ca. ‘*L. ossiferum’ depends on syntrophic interaction remains unknown. MK-D1^T^ and *Ca*. ‘L. ossiferum’ have similar cell morphologies, including long branching protrusions and blebs on the cell exterior [47].

**Fig. 2.**
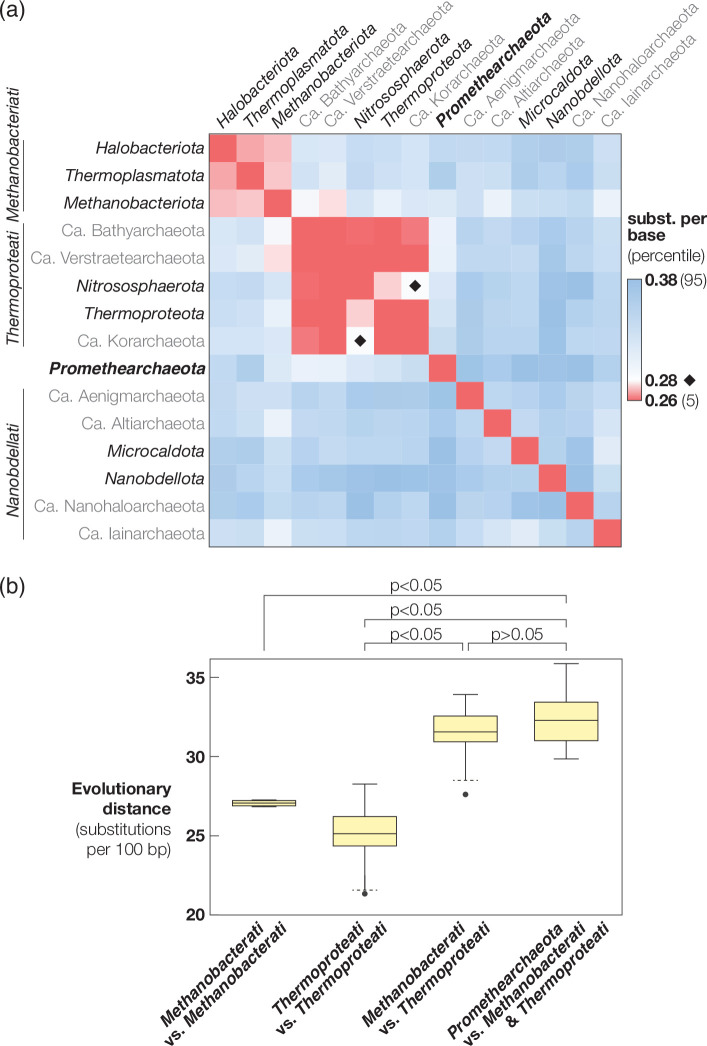
Evolutionary distances between archaeal phyla and kingdoms. (**a**) The average evolutionary distances (substitutions per 100 bases) between archaeal phyla are shown as a heatmap. For each phylum pair, the average evolutionary distance between representative sequences of each phylum (clustered based on 97 % sequence identity) was calculated using the silva r138.1 16S rRNA gene alignment. Sequences of members of the classes *Halobacteria* and *Thermoplasmata* were omitted from the analysis due to their long branches (see methods). The 5th and 95th percentiles and the maximum evolutionary distance among *Methanobacteriati*/*Thermoproteati* (indicated with diamonds) were used to define the heatmap scale. (**b**) Box plots of the evolutionary distances between/within kingdoms. Whiskers indicated with solid lines are based on maximum and minimum values. When outliers were present (1.5× interquartile range below the first quartile or above the third quartile), whiskers were drawn with a dotted line at 1.5× interquartile range below the first quartile or above the third quartile. Outliers are indicated by black dots. Statistical significance is indicated for comparison of the distances between/within kingdoms.

In accordance with ICNP guidelines and reflecting the physiological and phylogenetic characteristics of the organism, we propose a novel genus and species, *Promethearchaeum syntrophicum* gen. nov., sp. nov. to accommodate this isolate. So far *Promethearchaeum syntrophicum* and *Ca*. ‘L. ossiferum’ each respectively remain the only isolated and only enriched representatives of the lineage ‘Asgard’ archaea/‘Asgardarchaeota’, which lacks a formal phylum name that conforms to ICNP regulations [13,14]. Furthermore, this lineage is also distinct from other archaeal phyla/supergroups [3,30] that comprise the three archaeal kingdoms (Fig. 1), *i.e*., *Methanobacteriati*, *Thermoproteati*, and *Nanobdellati*, which have been proposed in accordance with the recent emendation of ICNP to include the category of kingdom [14]. Supporting the hypothesis that ‘Asgard’ archaea/‘Asgardarchaeota’ represent a distinct (*i.e*., fourth) kingdom, we find that the average evolutionary distances (based on the silva r138.1 16S rRNA gene alignment) between ‘Asgard’ archaea/‘Asgardarchaeota’ and *Methanobacteriati* or *Thermoproteati* are significantly different (*P*<0.05) from the distances within those kingdoms (>0.286 *vs*. <0.283 substitutions per base; *P*<0.05) and not statistically different from the distances between *Methanobacteriati* and *Thermoproteati* (>0.287 excluding one outlier; *P*>0.05) (Fig. 2). With recent rules set by the ICNP, phylum and kingdom names must be based on the name of the type genus (Rule 8), and when only one genus name is validly published under the ICNP, this genus must be designated as the type genus for each taxonomic category (Rule 22) [11,12]. The valid publication of the genus *Promethearchaeum*, as described below, would make the genus eligible for designation as the nomenclatural type for the higher taxa up to the kingdom rank: therefore, we also propose the associated family, order, class, phylum, and kingdom as *Promethearchaeaceae* fam. nov., *Promethearchaeales* ord. nov., *Promethearchaeia* class. nov., *Promethearchaeota* phyl. nov., and *Promethearchaeati* regn. nov. with *Promethearchaeum* as the type genus.

### Note

Rule 30(3)(b) of ICNP stipulates that the valid publication of a species name necessitates the deposition of a viable culture of a type strain of that species in at least two publicly accessible culture collections in different countries, with exceptions made for microorganisms that require specialised facilities (*e.g*., Risk Group/Biological Safety Level 3, high-pressure requirements, etc.) [12]. An illustrative case is *Pyrococcus yayanosii* strain CH1^T^ (JCM 16557). This strain has been deposited in only one culture collection because it requires specialised equipment for cultivation under high-pressure conditions, but it has been recognised as a species with a validly published name after discussion by a committee consisting of the Chairman of the International Committee on Systematics of Prokaryotes (ICSP), the Chairman of the Judicial Commission of the ICSP, and the Editor in Chief of the International Journal of Systematic and Evolutionary Microbiology [48]. We posit that MK-D1^T^ also merits consideration as an exception owing to its exceptionally low cell yield and the need to confirm growth through qPCR. Due to these challenges, only one publicly accessible culture collection (JCM) has successfully cultured and preserved MK-D1^T^. While efforts are currently underway to allow its deposition in another culture collection in a different country, the culture remains available upon request from the JAMSTEC culture collection as an alternative to the second publicly accessible culture collection.

While most validly published species names are based on isolates in axenic cultures, Rule 31a of the ICNP explicitly permits species description in regard with one member of unambiguous co-cultures (*e.g*., purified two-species cultures) [12]. An example cited in Rule 31a is *Syntrophobacter wolinii* Boone and Bryant 1984, which is described as one member of a syntrophic partnership with a hydrogen-consuming organism. Analogous instances can also be found in the genera *Pelotomaculum* [49,51], *Syntrophorhabdus* [52], *Microcaldus* [53], and *Nanobdella* [54]. Thus, the description of *Promethearchaeum syntrophicum* applied to one member of the pure obligately syntrophic co-culture with *Methanogenium* sp. strain MK-MG is legitimate.

A committee consisting of the Chair of the International Committee on Systematics of Prokaryotes (ICSP), the Chair of the Judicial Commission of the ICSP and the Editor-in-Chief of the International Journal of Systematic and Evolutionary Microbiology have granted an exception to ICNP Rule 30(3)(b) regarding deposition of a type strain in two recognised collections in two different countries. In addition to JCM, *Promethearchaeum syntrophicum* MK-D1^T^ is also available on request from the JAMSTEC culture collection under the DARWIN sample library (see below). Attempts are still under way to complete the deposition of the type strain in a second recognised culture collection in another country.

## Description of *Promethearchaeum* gen. nov.

*Promethearchaeum* (Pro.me.the.ar.chae’um. Gr. masc. n. *Prometheus,* the god who shaped humans out of mud and gave them the ability to create fire; N.L. neut. n. *archaeum*, an archaeon; N.L. neut. n. *Promethearchaeum*, an archaeon named for Prometheus).

Cells are small cocci, non-motile. Cells produce membrane vesicles, chains of blebs, and membrane-based protrusions. Only grows anaerobically and syntrophically with hydrogen- and formate-utilising microorganisms such as sulphate-reducing bacteria and methanogenic archaea. Chemoorganotrophic; utilises amino acids and peptides. Inhabits marine sediment.

The type species is *Promethearchaeum syntrophicum*.

## Description of *Promethearchaeum syntrophicum* sp. nov.

*Promethearchaeum syntrophicum* (syn.tro’phi.cum. Gr. prep. *syn*, in company with, together with; Gr. masc/fem. n. *trophos*, feeder, rearer, one who feeds; N.L. neut. adj. *syntrophicum*, syntrophic).

Strictly anaerobic. Cells are small cocci, approximately 300–750 nm in diameter (average, 550 nm), and generally form aggregates surrounded by EPS in the exponential growth phase. Produces membrane vesicles and chains of blebs. In the late exponential to stationary phases, cells produce membrane-based protrusions. The organism only grows syntrophically on amino acids/peptides with H_2_- and formate-utilising organisms. Growth occurs with casamino acids, powdered milk, casein digest, peptone, tryptone, and yeast extract. The following organic and inorganic substances are not utilised: H_2_, formate, glucose, fructose, xylose, ribose, maltose, lactate, acetate, citrate, pyruvate, fumarate, alginate, l-hydroxyproline, sulphate, thiosulphate, and nitrate. Temperature range for growth is between 4 and 30 °C with the optimum at 20 °C.

The type strain, MK-D1^T^ (=JCM 39240^T^), was isolated from deep-sea methane-seep sediment of the Nankai Trough at 2533 m water depth, off the Kumano area, Japan. The DNA G+C content of the type strain is 31.1 mol% (determined from the genome sequence). The type strain is also available by request from the JAMSTEC culture collection under the DARWIN sample library (number 115508) (https://www.godac.jamstec.go.jp/darwin/en/index.html).

The GenBank/EMBL/DDBJ accession numbers for the 16S rRNA gene and genome sequences of strain MK-D1^T^ are LC490619 and CP042905, respectively.

## Description of *Promethearchaeaceae* fam. nov.

*Promethearchaeaceae* (Pro.me.the.ar.chae.a.ce’ae. N.L. neut. n. *Promethearchaeum*, type genus of the family; suff. -*aceae*, ending to denote a family; N.L. fem. pl. n. *Promethearchaeaceae,* the family of the genus *Promethearchaeum*).

Description of the family *Promethearchaeaceae* is the same as for the genus *Promethearchaeum*. The type genus is *Promethearchaeum*.

## Description of *Promethearchaeales* ord. nov.

*Promethearchaeales* (Pro.me.the.ar.chae.a’les. N.L. neut. n. *Promethearchaeum*, type genus of the order; suff. -*ales*, ending to denote an order; N.L. fem. pl. n. *Promethearchaeales*, the order of the genus *Promethearchaeum*).

Description of the order *Promethearchaeales* is the same as for the genus *Promethearchaeum*. The type genus is *Promethearchaeum*.

## Description of *Promethearchaeia* class. nov.

*Promethearchaeia* (Pro.me.the.ar.chae’i.a. N.L. neut. n. *Promethearchaeum*, type genus of the class; suff. -*ia*, ending to denote a class; N.L. fem. pl. n. *Promethearchaeia*, the class of the genus *Promethearchaeum*).

Description of the class *Promethearchaeia* is the same as for the genus *Promethearchaeum*. The type genus is *Promethearchaeum*.

## Description of *Promethearchaeota* phyl. nov.

*Promethearchaeota* (Pro.me.the.ar.chae.o’ta. N.L. neut. n. *Promethearchaeum*, type genus of the phylum; suff. -*ota*, ending to denote a phylum; N.L. neut. pl. n. *Promethearchaeota*, the *Promethearchaeum* phylum).

Description of the phylum *Promethearchaeota* is the same as for the genus *Promethearchaeum*. Type genus is *Promethearchaeum*.

Originally detected through 16S rRNA gene clone library analysis by teams led by Ken Takai (JAMSTEC) and by Constantino Vetriani (Rutgers University) and termed Hydrothermal Vent Crenarchaeotic Group (DHVC1), Ancient Archaeal Group (AAG), Marine Benthic Group-B (MBG-B), Deep-Sea Archaeal Group (DSAG), Marine Hydrothermal Vent Group-2 (MHVG-2), and Marine Hydrothermal Vent Group-3 (MHVG-3). The first genome was acquired through metagenomics by a team led by Thijs J. G. Ettema (Uppsala University; now Wageningen University and Research) and Lionel Guy (Uppsala University), terming the group as ‘Lokiarchaeota’ (and later expanding the group and terming it ‘Asgard’ archaea) and discovering a new evolutionary connection between Archaea and Eukaryotes. Subsequent metagenomic and phylogenetic analyses have identified many class-level groups also belonging to the group ‘Asgard’ archaea: ‘Asgardarchaeia’, ‘Baldrarchaeia’, ‘Borrarchaeia’, ‘Heimdallarchaeia’, ‘Hermodarchaeia’, ‘Jordarchaeia’, ‘Odinarchaeia’, ‘Sifarchaeia’, ‘Thorarchaeia’, and ‘Wukongarchaeia’ (‘Lokiarchaeota’ was reclassified as a class-level lineage ‘Lokiarchaeia’).

## Description of *Promethearchaeati* regn. nov.

*Promethearchaeati* (Pro.me.the.ar.chae.a’ti. N.L. neut. n. *Promethearchaeum*, type genus of the kingdom; suff. -*ati*, ending to denote a kingdom; N.L. masc. pl. n. *Promethearchaeati*, the *Promethearchaeum* kingdom).

Description of the kingdom *Promethearchaeati* is the same as for the genus *Promethearchaeum*. Type genus is *Promethearchaeum*.

## supplementary material

10.1099/ijsem.0.006435 Uncited Supplementary Material 1.

## Abbreviations

AAG: ancient archaeal group
2-BES: 2-bromoethansulfonate
DHS: down-flow hanging sponge
DHVC1: deep-sea hydrothermal vent crenarchaeotic group
DSAG: deep-sea archaeal group
EPS: extracellular polymer substances
ESPs: eukaryotic signature proteins
FIB-SEM: focused ion beam-scanning electron microscopy
ICNP: International Code of Nomenclature of Prokaryotes
ICSP: International Committee on Systematics of Prokaryotes
MBG-B: marine benthic group-B
MHVG-2: marine hydrothermal vent group-2
MHVG-3: marine hydrothermal vent group-3
QFDE-EM: quick-freeze deep-etch replica electron microscopy
qPCR: quantitative polymerase chain reaction
S-layer: surface layer
TEM: transmission electron microscopy
