# Supplementary material for: Promethearchaeum syntrophicum gen. nov., sp. nov., an anaerobic, obligately syntrophic archaeon, the first isolate of the lineage ‘Asgard’ archaea, and proposal of the new archaeal phylum Promethearchaeota phyl. nov. and kingdom Promethearchaeati regn. nov
Source: Int J Syst Evol Microbiol. 2024 Jul 5;74(7):006435. doi: 10.1099/ijsem.0.006435 (PMC11316595; doi:10.1099/ijsem.0.006435)
Supplement: Uncited Supplementary Material 1. [file ijsem-74-06435-s001.pdf]

International Journal of Systematic and Evolutionary Microbiology

Supplementary material

Promethearchaeum syntrophicum gen. nov., sp. nov., an anaerobic, obligately syntrophic archaeon, the first isolate of the lineage 'Asgard' archaea, and proposal of the new archaeal phylum *Promethearchaeota* phyl. nov. and kingdom *Promethearchaeati* regn. nov.

Hiroyuki Imachi[1]*, Masaru K. Nobu[1]*, Shingo Kato[2,3], Yoshihiro Takaki[1], Masayuki Miyazaki[1], Makoto Miyata[4], Miyuki Ogawara[1], Yumi Saito[1], Sanae Sakai[1], Yuhei O. Tahara[4], Yoshinori Takano[5], Eiji Tasumi[1], Katsuyuki Uematsu[6], Toshihiro Yoshimura[5], Takashi Itoh[2], Moriya Ohkuma[2], and Ken Takai[1,7]

[1]Institute for Extra-cutting-edge Science and Technology Avant-garde Research (X-star), Japan Agency for Marine-Earth Science and Technology (JAMSTEC), Yokosuka, Japan
[2]Japan Collection of Microorganisms (JCM), RIKEN BioResource Research Center, Tsukuba, Japan
[3]Submarine Resources Research Center, JAMSTEC, Yokosuka, Japan
[4]Graduate School of Science, Osaka Metropolitan University, Osaka, Japan
[5]Biogeochemistry Research Center, JAMSTEC, Yokosuka, Japan
[6]Department of Marine and Earth Sciences, Marine Work Japan, Yokosuka, Japan
[7]Section for Exploration of Life in Extreme Environments, Exploratory Research Center on Life and Living Systems (ExCELLS), National Institute of Natural Sciences, Okazaki, Japan

Corresponding authors:
Hiroyuki Imachi, E-mail: imachi@jamstec.go.jp
Masaru K. Nobu, E-mail: mnobu@ jamstec.go.jp

Supplementary Table S1. Relative abundance of strain MK-D1^T, *Halodesulfovibrio* sp. strain MK-HDV and *Methanogenium* sp. strain MK-MG during/after construction of the pure co-culture of strain MK-D1^T and *Halodesulfovibrio* sp. strain MK-HDV. Each transferred culture was incubated for approximately 90–120 days and subjected to DNA extraction, qPCR and 16S rRNA gene tag-sequencing. The two columns on the right show the DNA concentration extracted from 1 ml of culture liquid and the 16S rRNA gene copy number for strain MK-D1^T after cultivation. ND, not detected. The 16S rRNA gene tag-sequence data was deposited in PRJDB17953 with Biosample accession numbers SAMD00769478–SAMD00769486.

Number of successive transfer	Culture incubation time (days)	Sequence read numbers	Relative abundance (%)			DNA conc. (ng/μL)*	16S rRNA gene copies of MK-D1 (copies/ml)
			MK-D1	Halodesulfovibrio MK-HDV	Methanogenium MK-MG		
1	86	83487	25.1	72.4	2.5	1.55	1.06E+06
2	90	84385	29	70.7	0.3	1.2	1.88.E+06
3	92	154313	28.4	71.5	0.1	1.04	2.04E+06
4	101	156046	18.5	84.5	0.01	1.35	1.66E+06
5	105	118210	24.1	75.9	ND	0.94	3.39E+06
6	104	115549	21.2	78.8	ND	1.62	3.45E+06
7	104	91250	26.2	73.8	ND	1.56	1.26E+06
8	110	91072	15.3	84.7	ND	2.26	7.96E+05
9	122	64058	17.4	82.6	ND	1.19	1.40E+06

*Extracted DNAs were dissolved in 21 μL TE buffer. The DNA concentrations were quantified by Quant-iT dsDNA High-Sensitivity Assay Kit (Qiagen).

Supplementary Table S2. Amino acid concentrations during the growth of strain MK-D1^T in pure co-cultures in the anaerobic medium supplemented with casamino acids (0.05%, w/v) and powdered milk (0.05%, w/v). The concentrations of amino acids were quantified and presented as relative concentrations, normalized to an initial value set at 100% at 0 day of the cultivation experiment. Abbreviations for Ala (alanine), Gly (glycine), Val (valine), Leu (leucine), Ile (isoleucine), Pro (proline), Asp (aspartic acid), Thr (threonine), Ser (serine), Met (methionine), Glu (glutamic acid), Phe (phenylalanine), Hyp (hydroxyproline), and Tyr (tyrosine) in the order of gas chromatographic determination. The combined signals from glutamate (Glu, glutamic acid; Gln, glutamine) and aspartate (Asp, aspartic acid; Asn, asparagine) are shown as Glu and Asp, respectively, because of the acid hydrolysis treatment. A part of the amino acid concentration data and growth curves of strain MK-D1^T have been reported by Imachi et al. 2020. Nature. 577: 519–525 (see Extended Data Fig. 1b and c).

		Culture #1		Culture #2	
	0 day	45 days	90 days	45 days	90 days
Ala	100.0	87.0	42.7	112.7	74.3
Gly	100.0	95.1	59.7	95.6	77.2
Val	100.0	95.6	49.5	105.6	76.3
Leu	100.0	99.8	63.4	95.5	79.2
Ile	100.0	98.5	58.4	91.0	74.1
Pro	100.0	88.2	75.7	102.2	81.5
Asp	100.0	102.6	91.8	107.0	101.0
Thr	100.0	113.6	89.7	99.8	91.2
Ser	100.0	112.4	90.8	108.5	100.9
Met	100.0	113.9	103.0	106.9	90.2
Glu	100.0	102.6	95.1	107.9	102.2
Phe	100.0	103.6	97.8	102.1	98.6
Hyp	100.0	98.9	130.2	98.4	89.0
Tyr	100.0	107.6	109.5	114.5	95.2

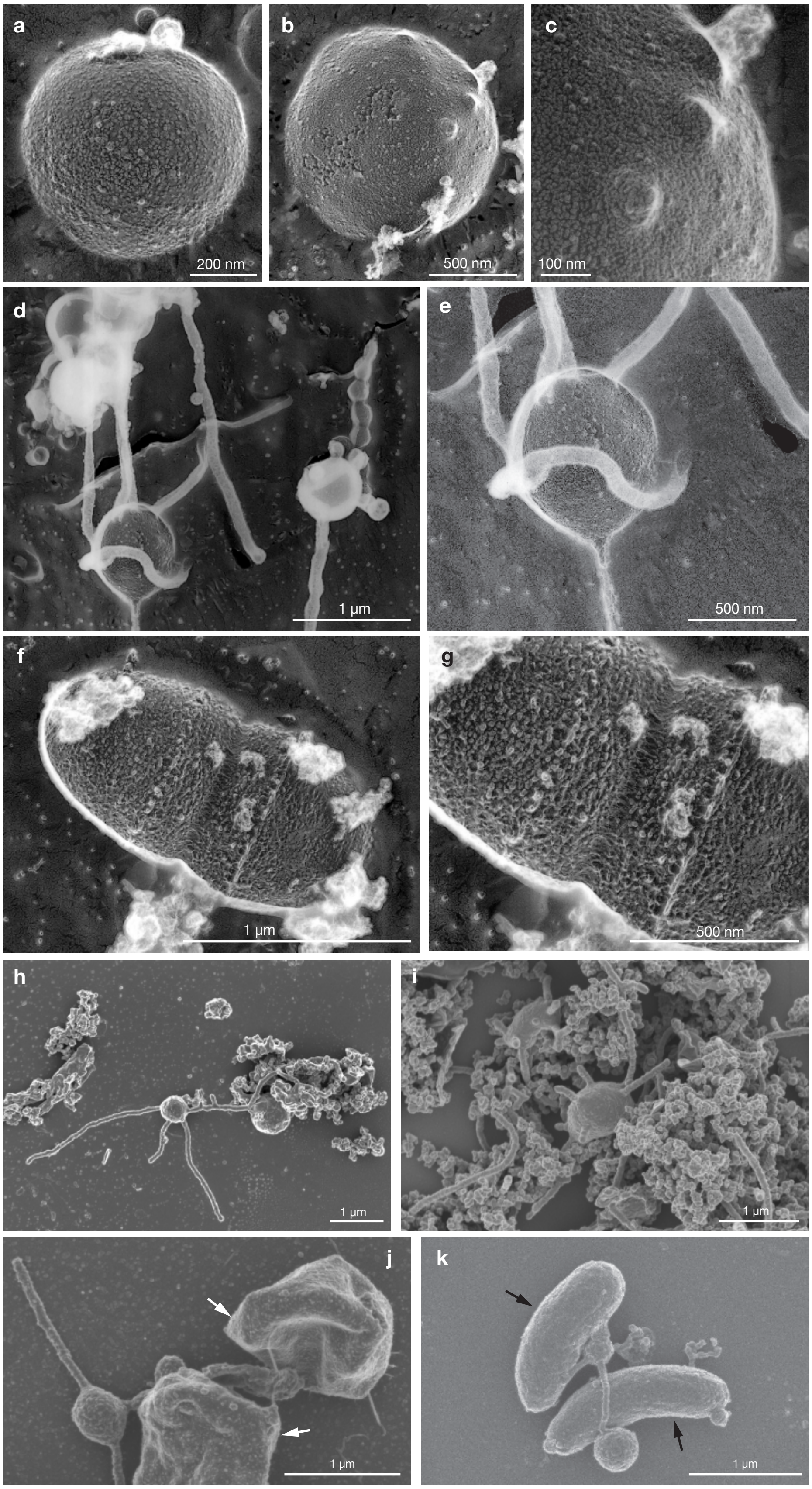

Supplementary Fig. S1. Photomicrographs of strain MK-D1^T. (a–g) QFDE-EM images of strain MK-D1^T. Single cells (a–c). The image (c) shows a magnified image of (b). Cells producing protrusions (d and e). The image (e) shows a magnified image of (d). The protrusions appear white because platinum is also deposited on the backside. A dividing cell (f and g). The image (g) shows a magnified image of (f). (h–k) Scanning electron microscopic images of strain MK-D1^T. Cells producing protrusions (h and i). (i and j) Syntrophic associations of strain MK-D1^T and hydrogen/formate-utilizing microorganisms. Strain MK-D1^T with *Methanogenium* sp. strain MK-MG (j) or *Halodesulfovibrio* sp. MK-HDV (k). White and black arrows indicate *Methanogenium* and *Halodesulfovibrio* cells, respectively. The images (a–c, f, and g) and images (d, e, h–k) were taken from cultures in the middle exponential phase (i.e., 80 days of incubation) and in the late exponential phase (i.e., 105 or 110 days of incubation), respectively.

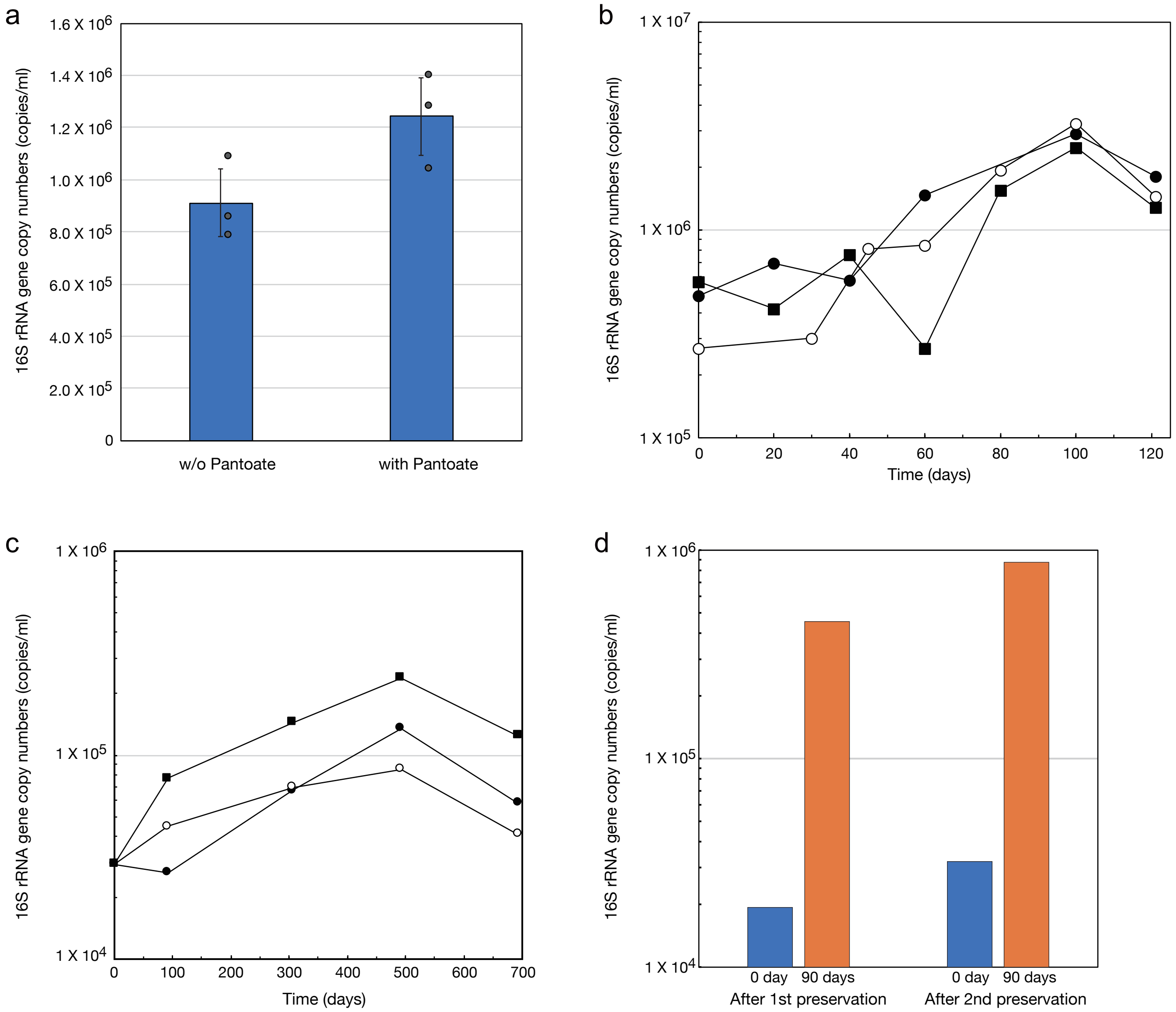

Supplementary Fig. S2. Growth properties of strain MK-D1^{T}. (a) Effect of pantoate on growth of strain MK-D1^{T}. Data are mean ± standard deviations of triplicate determinations. Each data point is shown as a dot. Cultivation temperature was set at 20°C, the optimum growth temperature for the strain. (b) Growth curves of strain MK-D1^{T} grown on anaerobic medium supplemented with yeast extract and pantoate. (c) Growth curves of strain MK-D1^{T} at 4°C. The experiment was initiated by simultaneously inoculating three fresh media from one parent culture. Therefore, the 16S rRNA gene copy number of day 0 was calculated based on that of the inoculum culture. (d) Confirmation of growth strain MK-D1^{T} after the glycerol storage by qPCR. Culture liquid samples were taken on days 0 and 90 of incubation and qPCR was performed.

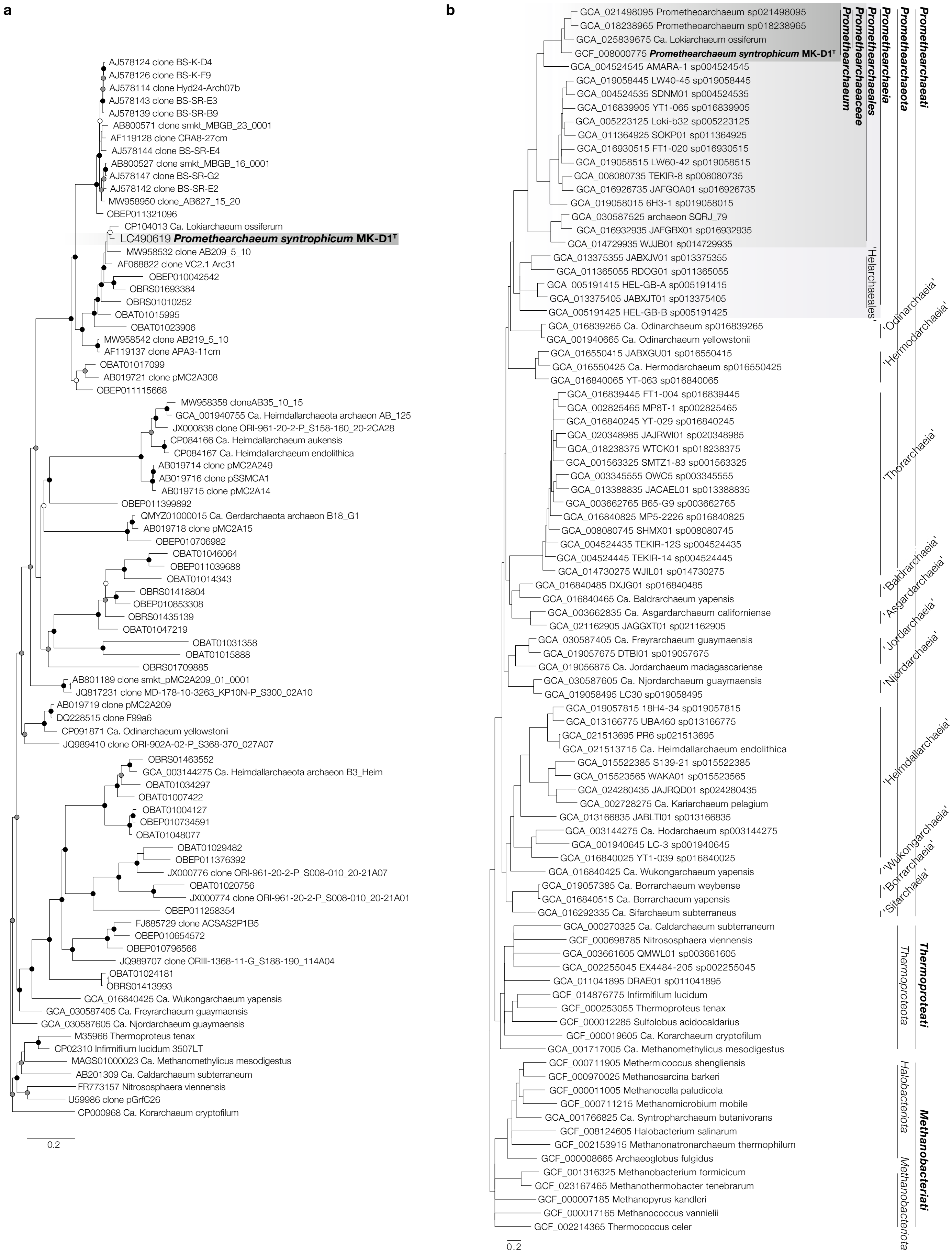

Supplementary Fig. S3. Phylogeny of *Promethearchaeum syntrophicum* strain MK-D1^T (unclasped version of Figure 1). (a) Phylogenetic tree of the 'Asgard' archaea group based on 16S rRNA genes. (b) Phylogenetic tree of the 'Asgard' archaea group based on a concatenated alignment of a subset of conserved marker proteins defined in GTDB r214.
